# Natural variation in wild tomato trichomes; selecting metabolites that contribute to insect resistance using a random forest approach

**DOI:** 10.1186/s12870-021-03070-x

**Published:** 2021-07-02

**Authors:** Ruy W. J. Kortbeek, Marc D. Galland, Aleksandra Muras, Frans M. van der Kloet, Bart André, Maurice Heilijgers, Sacha A. F. T. van Hijum, Michel A. Haring, Robert C. Schuurink, Petra M. Bleeker

**Affiliations:** 1 Green Life Science Research Cluster, Swammerdam Institute for Life Sciences, University of Amsterdam, 1098 XH Amsterdam, The Netherlands; 2 Data Analysis Group, Swammerdam Institute for Life Sciences, University of Amsterdam, 1098 XH Amsterdam, The Netherlands; 3 Enza Zaden Research & Development B.V, Haling 1E, 1602 DB Enkhuizen, The Netherlands; 4 Radboud University Medical Center, Bacterial Genomics Group, Geert Grooteplein Zuid 26-28, 6525 GA Nijmegen, The Netherlands

**Keywords:** Tomato, Trichomes, Specialised metabolites, Insect resistance, Whitefly, Thrips, Random forest, Volatiles, Acylsugars

## Abstract

**Background:**

Plant-produced specialised metabolites are a powerful part of a plant’s first line of defence against herbivorous insects, bacteria and fungi. Wild ancestors of present-day cultivated tomato produce a plethora of acylsugars in their type-I/IV trichomes and volatiles in their type-VI trichomes that have a potential role in plant resistance against insects. However, metabolic profiles are often complex mixtures making identification of the functionally interesting metabolites challenging. Here, we aimed to identify specialised metabolites from a wide range of wild tomato genotypes that could explain resistance to vector insects whitefly (*Bemisia tabaci*) and Western flower thrips (*Frankliniella occidentalis)*. We evaluated plant resistance*,* determined trichome density and obtained metabolite profiles of the glandular trichomes by LC-MS (acylsugars) and GC-MS (volatiles). Using a customised Random Forest learning algorithm, we determined the contribution of specific specialised metabolites to the resistance phenotypes observed.

**Results:**

The selected wild tomato accessions showed different levels of resistance to both whiteflies and thrips. Accessions resistant to one insect can be susceptible to another. Glandular trichome density is not necessarily a good predictor for plant resistance although the density of type-I/IV trichomes, related to the production of acylsugars, appears to correlate with whitefly resistance. For type VI-trichomes, however, it seems resistance is determined by the specific content of the glands. There is a strong qualitative and quantitative variation in the metabolite profiles between different accessions, even when they are from the same species. Out of 76 acylsugars found, the random forest algorithm linked two acylsugars (S3:15 and S3:21) to whitefly resistance, but none to thrips resistance. Out of 86 volatiles detected, the sesquiterpene α-humulene was linked to whitefly susceptible accessions instead. The algorithm did not link any specific metabolite to resistance against thrips, but monoterpenes α-phellandrene, α-terpinene and β-phellandrene/D-limonene were significantly associated with susceptible tomato accessions.

**Conclusions:**

Whiteflies and thrips are distinctly targeted by certain specialised metabolites found in wild tomatoes. The machine learning approach presented helped to identify features with efficacy toward the insect species studied. These acylsugar metabolites can be targets for breeding efforts towards the selection of insect-resistant cultivars.

**Supplementary Information:**

The online version contains supplementary material available at 10.1186/s12870-021-03070-x.

## Background

Pests and diseases are a threat for food security with yearly losses of around 26% in primary yield, and even higher secondary yield losses up to 38% [1]. Herbivorous insects can damage plants directly through feeding, resulting in decreased photosynthetic capacity and in visible damage rendering crop plants unmarketable. However, they can also be vectors of diseases, typically transferring viruses from plant to plant, providing the virus mobility that can dramatically lower production. Insects like whiteflies (*Bemisia tabaci*; previously Sweet Potato- or Silverleaf Whitefly) and western flower thrips (*Frankliniella occidentalis*) are important, invasive herbivores found in agricultural production areas all over the world. Both insect species are regarded “supervectors” that can carry and transmit a multitude of viruses to a large range of host plants by feeding on the phloem (whitefly) or cell content (thrips) [2–5]. For both insects, plant-to-plant virus transmission predominantly takes place during their (mobile) adult life phase [6, 7]. For thrips, however, the virus is acquired exclusively in larval stage prior to developing into an infectious adult vector [8, 9]. Management of these pest vectors in agriculture is still largely based on chemical control and systemic insecticides that can provide a strong and lasting crop protection. However, with an increasing number of classical synthetic insecticides banned due to their negative off-target effects, understanding and deploying the insect resistance found in (wild) ancestral crop relatives is again gaining interest [10–13].

Plant resistance against (herbivorous) insects can be defined as a heritable ability to decrease insect attack in comparison to other plants without this ability thereby lowering inflicted damage [14, 15]. In nature, plants reduce or prevent insect colonisation through defence mechanisms such as anti-settling or anti-feeding properties, toxicity to the insect or interference in its development [16–18]. They range from physical barriers, surface coverage with sticky substances or the production of enzymes and specialised metabolites. Whereas non-glandular hairs physically impede the movement of certain herbivores on the plant surface, glandular tissues such as ducts, peltate cells or, in the case for tomato, glandular trichomes, produce and store specialised metabolites that act in defence against insects [19, 20]. Metabolites stored inside such glandular tissues are released via exudation or by disruption of the tissue through physical damage by the insect (e.g. chewing, penetration or touch). The exact mode of action of the phytochemicals is generally not fully elucidated. Besides physical impediments like stickiness or clogging of the insect’s mouthparts, specialised metabolites can act on the molecular level by receptor interference, disturbance of biological (e.g. enzymatic) processes, hormone balance or affect the insect’s nervous system by interfering in electron transport [21].

Tomato produces a plethora of specialised metabolites in its glandular trichomes which are mainly located on the green parts of the plant. Especially certain wild relatives of cultivated tomato (*Solanum lycopersicum*) display considerable resistance to agronomically important pest insects, which often can be traced back to trichome-borne specialised-metabolites [22–27]. Wild species like *S. pennellii*, *S. habrochaites* and *S. galapagense*, for example, exude a broad range of acylsugars from type I- and IV-glandular trichomes providing resistance against several pest insects [28–30]. These acylsugars consist of glucose-or sucrose moieties with acyl groups esterified to one-or more of its hydroxyl groups. The composition of acylsugars exudates can be highly diverse, as the complete blend is an assembly of acylsugars having different sugar moieties and varying acyl chain length-and positions [31–35]. Interestingly, insects appear to respond differently to particular acylsugar compositions, indicating that insect targeting is component specific [36, 37].

The (semi-)volatile mono-and sesquiterpenes that are, at least predominantly, produced and stored in type-VI trichomes serve as another important class of metabolites implicated in the defence against pests [24, 38–40]. The sesquiterpenes 7-epizingiberene and R-curcumene produced by wild tomato *S. habrochaites* PI127826 repel whiteflies and 7-epizingiberene alcohol derivatives have a repellent effect on the spider mite *Tetranychus urticae* [41, 42]. Also, 2,3-dihydrofarnesoic acid from *S. habrochaites* accessions LA1363 and LA1927 act on spider mites as a repellent [43] and type-VI trichomes of *S. habrochaites* accession LA1777 produce a mixture of bergamotoic-and santanaloic acids that confers resistance against the tomato fruitworm (*Helicoverpa zea*) and the beet armyworm (*Spodoptera exigua)* [44]. Type-VI trichomes store volatiles as concentrated oily substances in their internal storage-cavity, of which it is hypothesized that only a limited amount is released by (passive) diffusion [45]. Upon rupture, e.g. by touch, the insect receives a high dose of these essential oils which can be toxic by itself or creates toxic fumes [46, 47]. The biochemical (precursor-) pathways to produce these metabolites are rather conserved among tomato species, including cultivated tomato. As the leaves and stem surface of cultivated tomato also have type-VI and, although only in an early life stage, type-IV trichomes, the transfer of trichome-borne specialised metabolic-pathways from wild tomato to cultivars is feasible [19, 24, 48].

Although it is clear that metabolite-based insect-resistance traits are available from natural resources, it is still a challenge to identify those metabolites that target the pest insect of interest. The complexity in the blend of metabolites produced makes it difficult to identify the functional compound(s) causal to an observed phenotype (e.g. insect mortality or arrested development). Machine learning methodologies, including Random Forest (RF) classification, have emerged as powerful and accurate alternatives to the often-used dimension-reduction methods (e.g. Partial Least Squares regression) to determine variables discriminating (phenotypic) classes [49, 50]. Examples of successful RF applications in plant biology include the selection of metabolic markers for drought tolerance in potato [51] and the identification of DNA-methylation patterns in maize determining gene transcription-and translation [52]. The ability of RF to cope with large amounts of “omics” data derived from a relatively small number of samples makes RF also a promising tool to identify anti-insect metabolites from natural resources.

Here, we explore the natural variation in resistance to insects (i.e. whitefly and thrips) and production of specialised metabolites by glandular trichomes within a panel of wild tomato accessions. To this end we successively quantified insect survival, density of different glandular trichome-types, and production of acylsugars and trichome-borne volatiles in the accessions. Next, we used a customised RF algorithm to predict which acylsugars and volatile molecules correlated with survival phenotypes of the insects. This resulted in a list of metabolites predicted to be involved in insect-specific resistance or susceptibility. Finally, the biological outcome, as well as the use of RF classification models on this type of metabolite-phenotype data, will be discussed.

## Results

### Differential insect responses on tomato accessions

**Fig. 1:**
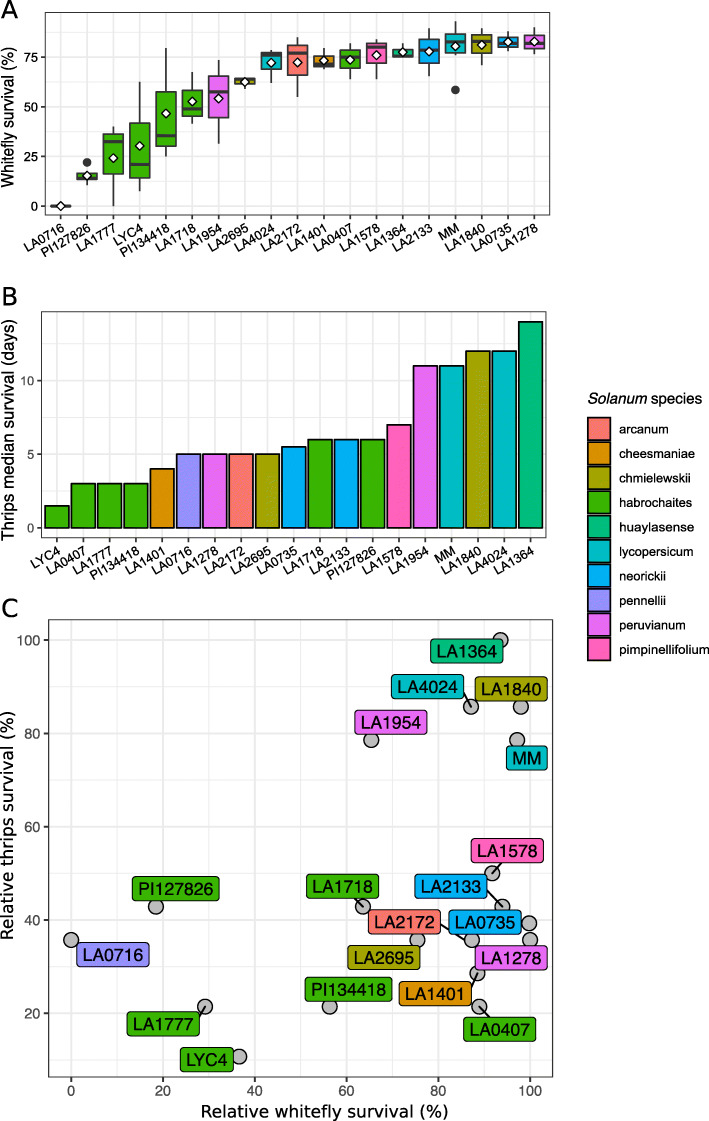
Insect response to selected *Solanum* accessions (**a**) Adult whitefly (*B. tabaci*) survival after 5 days in clip-cages attached to leaflets of the fourth fully expanded leaf from the top of six-weeks-old plants (*n* = 3–8). Boxplots indicate mean (diamonds) and median (black horizontal bars) survival rates. Accessions are ordered by ascending mean survival rates. **b** Median survival time (days) of thrips L1 larvae (*F. occidentalis*) placed on a leaf disc (*n* = 24–36) for a maximum of 19 days. Leaf discs were made from the fourth fully expanded leaf from the top of six-week-old plants. Accessions are ordered by ascending median survival. **c** Relative survival scores of whiteflies and thrips plotted against one another. Relative survival score was calculated by setting the highest mean (whitefly) or median (thrips) survival rate to 100%. Accessions are colour-coded by their *Solanum* species

Survival of whitefly was assessed on different tomato accessions in a no-choice set up. Survival ranged from zero on *S. pennellii* accession LA0716, to 88% on *S. peruvianum* LA1278 (Fig. 1a). In addition, also the performance of thrips (*F. occidentalis*) on the same set of wild tomato was determined in a different survival assay using thrips larvae; the life stage in which thrips acquires viruses. In this experimental setup, the larval survival on each accession was recorded for 15 days on leaf discs whereafter the median survival time (i.e. when 50% of the thrips larvae deceased) was estimated using the Kaplan-Meier method giving the most conclusive proxy for the phenotype (Additional file: Figure S1). This resulted in a different ranking of the accessions than for whitefly survival, with median survival ranging from 1.5 days on *S. habrochaites* LYC4 to 14 days on *S. huaylense* LA1364 (Fig. 1b). These differences were further visualised when the survival parameters were plotted against each other (Fig. 1c). It showed that although some accessions were indeed a toxic environment to both insects (e.g. *S. habrochaites* LA1777), this was not the case for all. For example, whiteflies had a relative high survival rate on LA0407 and LA1278, whereas thrips did not. PI127826, on the other hand, was resistant to whiteflies but marginally more susceptible to thrips. As expected, the *S. lycopersicum* accessions Moneymaker (MM) and LA4024 benchmark susceptibility to both insects.

### Trichome phenotype of the selected genotypes

**Fig. 2:**
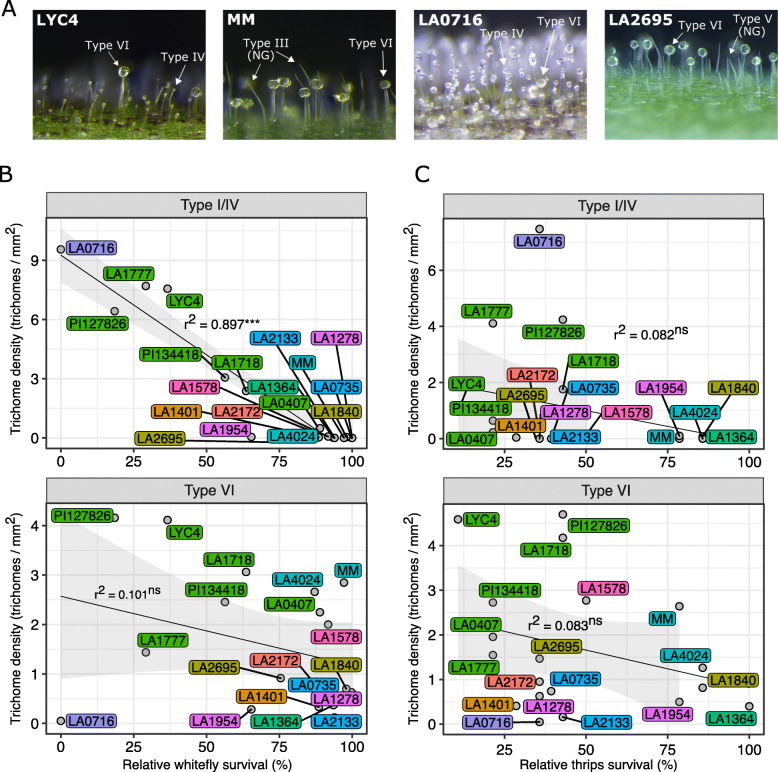
Insect survival plotted against glandular-trichome densities on leaves of selected accessions. **a** Photographs of *S. habrochaites* LYC4, *S. lycopersicum* cv. Moneymaker (MM), *S. pennellii* LA0716 and *S. chmielewskii* LA2695 to visualise the diversity in trichome-types. Images of all accessions can be found in Additional file: Figure S2.**b** Mean glandular trichome density per mm^2^ leaf surface of type I/IV trichomes (top panel) and type VI trichomes (lower panel) against relative whitefly survival rates on the different accessions and **c** against thrips (*F. occidentalis)* relative survival. The black regression lines show the linear relationship, with the 95% confidence interval in light grey, between the trichome densities and the survival phenotypes. The *r*^2^ is given together with asterisks for the significance of the relationships with *p*-values < 0.05*, < 0.01**, < 0.001***, ns: non-significant. Leaf material analysed originated from the fourth fully expanded leaf from the top of six-week-old plants

Following the hypothesis that the antixenosis phenotype is facilitated by trichome-produced specialised metabolites, we first profiled the trichome density for each accession (illustrated in Fig. 2a, for all accessions see Additional file: Figure S2). Leaf-trichome density was determined for different trichome types: non-glandular (NG; including trichome-type II/III/V/VIII), glandular type I/IV and glandular type VI (Additional file: Figure S3). Comparing the trichome densities on the abaxial-and adaxial leaf surfaces shows that especially type I/IV trichomes prevail on the abaxial leaf surface. Type VI trichomes appear to be more or less equally distributed across adaxial- and abaxial leaf surfaces, with the exceptions of LA2695, LA1578 and LA1718 that have more type-VI trichomes on the adaxial-leaf surface. *S. pimpinellifolium* LA1578 is the hairiest accession in the panel with the majority of trichomes being NG.

To visualise the relationship between glandular-trichome density and insect response, we plotted the type-I/IV and type-VI trichome density of each accession against the insect survival rates (Fig. 2b, c). A higher density of type I/IV trichomes, typically found on *S. pennellii* LA0716 and *S. habrochaites* LA1777, LYC4, PI127826 and PI134418 accessions (Additional file: Figure S3), appeared to correlate to lower whitefly survival (Fig. 2b, top panel; *r*^2^ = 0.897, *p* > 0.001). In contrast, we did not find a significant linear relationship between type-I/IV density and the survival of the thrips larvae (Fig. 2c top panel; *r*^2^ = 0.082, *p* = 0.044). Also, the linear regression models did not indicate a linear relationship between type-VI trichome density and the survival rates of either whiteflies or thrips (Fig. 2b, c, lower panels; *p* > 0.05). For example, with approximately 300 type-VI trichomes per cm^2^, *S. lycopersicum* cultivars MM and LA4024 were amongst the most densely type-VI haired accessions (Additional file: Figure S3) but displayed high insect survival rates. Additional analysis on NG trichomes, and all trichome types combined, also did not yield a significant relationship between densities and the survival rates of either of the insects (Additional file: Figure S3).

### Specialised metabolite composition

Although glandular trichomes can contain other specialised metabolites, we focussed here on the most abundant molecules in type I/IV and type VI glandular trichomes: i.e. acylsugars and volatile molecules respectively. Acylsugars were obtained by leaf washes and analysed by liquid-chromatography mass-spectrometry (UHPLC-MS). Untargeted analysis resulted in a list of 76 acylsugar moieties across the 19 tomato accessions that were annotated according to their sucrose (S) or glucose (G) backbone, followed by the number of acyl chains plus the total number of carbon atoms distributed over the acyl chains. Reflecting the type-I/IV trichome density, accessions most resistant to whiteflies contain high amounts of acylsugars, but this was not necessarily the case for the accessions resistant to thrips (Additional file: Figure S4a-c). Although the majority of the detected acylsugars consist of acylsucroses, there was no indication that the type of backbone, i.e. sucrose or glucose, correlated to the levels of resistance. However, acylsugar exudates of whitefly resistant accessions consisted mainly of tri-acylsugars (i.e. G3 and S3) whereas the more susceptible accessions, albeit in low levels, contained mostly tetra-and penta-acyl sugars (Additional File: Figure S4d). The total number of carbons atoms distributed over the acyl chains predominantly ranged from 14 to 21 in resistant accessions, and from 21 to 25 in susceptible plants (Additional File: Figure S4e).

**Fig. 3:**
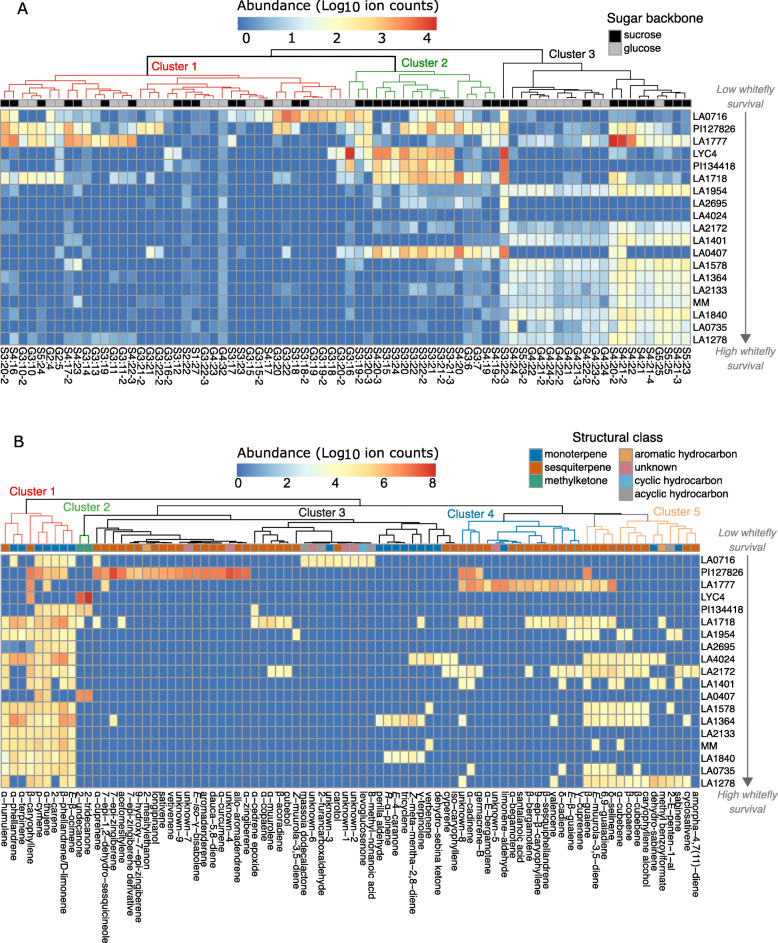
Heatmaps showing the detected metabolites in the different accessions. Accessions are ordered according to the mean whitefly survival rates and metabolites are clustered by complete-linkage clustering based on their abundance in the accessions. **a** Acylsugars detected by UHPLC-MS (*n* = 6) labelled using the following nomenclature: Sugar moiety backbone (G for glucose; S for sucrose) followed by the numbers of esterified acyl groups and the number of carbon atoms distributed over the acyl groups. In case of structural isomers, a hyphen followed by the isomer number is added. The panel on top of the heatmap indicates the sugar-moiety constituting the acylsugar backbone. **b** Volatiles detected by GC/MS-TOF (*n* = 3–4). Volatiles are labelled according to mass spectral matches to available libraries. The panel on top indicates the structural classification of the respective metabolite. Leaf material analysed originated from the fourth fully expanded leaf from the apex of six-week-old plants

To visualise individual acylsugars, we created a heatmap and clustered the acylsugars based on their abundance in different accessions ranked by whitefly survival (Fig. 3a). Form the appearing clusters, cluster 1 covers a selection of mainly acylglucoses that occurred only in a few resistant accessions. In this cluster, *S. pennellii* accession LA0716 contained a unique subset of acyl- glucoses and sucroses, while two *S. habrochaites* accessions contained another unique subset also comprising mixed set of sugar backbones. Cluster 2 compounds, mainly tri-acylsucroses (S3), were overrepresented in resistant *S. habrochaites* accessions. In susceptible accessions, acylsugars from cluster 3 were predominantly present, which mainly consisted of tetra-acylsucroses (S4) and tetra-acylglucoses (G4).

Besides acylsugars, trichome-derived volatiles were obtained by leaf washes and analysed by gas-chromatography mass-spectrometry (GC/MS-TOF). We detected 86 different volatiles in total and deduced their identity by comparison of their mass spectrum combined with their Kovats Retention Index (KI) to available libraries (Additional file: Table S3). The accessions displayed a diverse set of volatile profiles in which methyl ketones, monoterpenes, sesquiterpenes and other aromatic hydrocarbons were most abundant (Additional file: Fig. S5a). We did not find one of these structural classes to be predominantly present in the volatile profile of whitefly-resistant accessions, although the profile of susceptible accessions encompassed mainly monoterpenes (Additional File: Fig. S5a). Summing all ion-abundances per sample illustrates that neither whitefly nor thrips survival rates could be explained by the total level of volatiles detected in each accession (Additional File: Fig. S5b, c).

Plotting the individual metabolites resulted in a heatmap with dispersed abundances, visualising the rather distinct volatile profiles between accessions (Fig. 3b). Accessions with high whitefly survival rates appeared to contain volatile metabolites annotated in cluster 1 and 5, i.e. predominantly mono-and sesquiterpenes, although these compounds were also found in accessions with low whitefly survival. The three most resistant accessions all had a unique set of metabolites. *S. pennellii* LA0716 for instance produced a distinct set of volatile compounds, annotated in cluster 3, constituting of cyclic-and acyclic (oxygenated) hydrocarbons whereas *S. habrochaites* PI127826 and LA1777 each display a distinct set of sesquiterpenes, annotated in cluster 2 and 4 respectively. Other *S. habrochaites* accessions, LYC4, PI134418 and LA1718, produced methyl ketones as indicated in cluster 2.

### Linking specialised metabolites to whitefly resistance

**Fig. 4:**
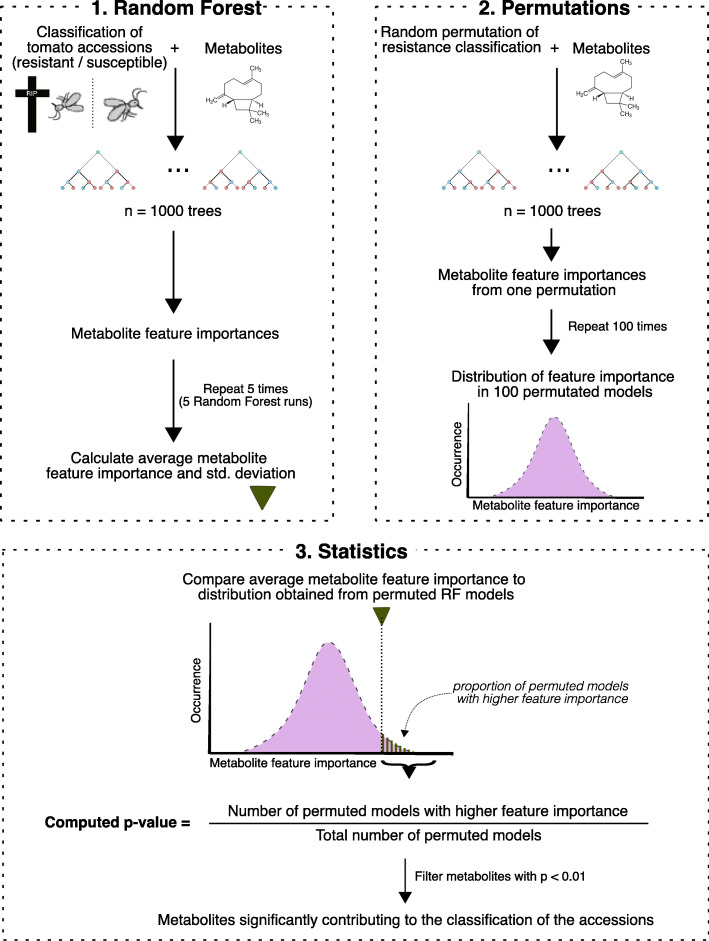
A schematic overview of the random forest analysis and metabolite selection procedure. First, (1) the RF algorithm was ran using resistant/susceptible labels as sample classifications and the metabolites as classifiers. This generates a feature importance for each metabolite and the average feature importance was calculated over 5 RF models. Next, (2) the sample classifications were randomly permutated over 100, 250 and 500 RF models, creating random feature-importance distributions per metabolite. Finally, (3) for each metabolite, the average feature importance calculated in (1) was compared to the distributions of (2) and *p*-values were calculated. Metabolites having an average feature importance significantly higher than their permuted models (*p* < 0.01) were predicted to contribute significantly to the resistant/susceptible classification of the accessions

**Fig. 5:**
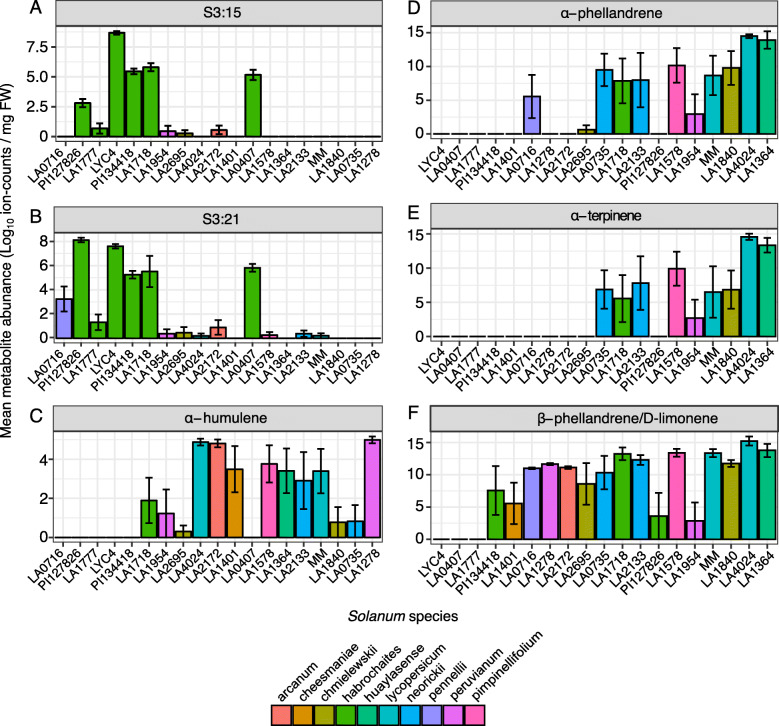
Acylsugars and volatiles contributing to the classification of the resistance phenotype of accessions. Plots show the abundance of the metabolites that were selected by the random forest algorithm to contribute to the resistant/susceptible classification of the accessions. Acylsugars (**a**, **b**) and volatiles (**c**) predicting the classification with respect to whitefly resistance. Accessions are ordered from low to high whitefly survival. **d**-**f** Volatiles predicting the classification with respect to thrips resistance. Accessions are ordered by ascending thrips survival medians. Bars represent log_10_-scaled mean ion-counts ± SE of the parent ion (acylsugars; *n* = 6) or base-peak (volatiles; *n* = 3–8)

To predict which specific, individual metabolites could be linked to the observed resistance phenotypes of the accessions, a Random Forest machine learning approach was applied. The RF model requires a binary classification of the accessions as an input variable and we therefore first fitted a generalised linear model (GLM) on the whitefly and thrips survival data creating two phenotypic classifications per insect proxy (Methods; Additional file: Table S1). The group of accessions with the lowest survival rates were hence labelled “resistant” while the remaining accessions were labelled “susceptible” (Table 1). As illustrated in Fig. 4, the RF algorithm was used to compute the importance of each metabolite (“metabolite feature-importance”) to reconstruct the phenotypic classification of the accessions as predefined by the GLM (see also: Methods). We first implemented the model on the acylsugars and the whitefly resistance classification of the accessions. The model exhibited a total accuracy score of 79%. We opted to apply a stringent *p*-value (*p* < 0.01) to the model resulting in two specific acylsugars, S3:15 and S3:21 (Table 2), that were identified as metabolites with significantly higher feature importances compared to the randomly permutated model (Additional file: Figure S6a, b), indicating their importance to separate the insect-phenotypic classes. Plotting the abundances of these two compounds indicates their predominantly presence in accessions classified as whitefly resistant. Acylsugar S3:15 occurred in *S. habrochaites* accessions PI127826, LYC4, PI134418, LA1718 while acylsugar S3:21 was also present in *S. pennellii* LA0716 (Fig. 5a, b). One susceptible *S. habrochaites* accession, LA0407, however also appeared to also produce both S3:15 and S3:21.

**Table 1:** List of 19 selected *Solanum* accessions and their classification

Accession	Species	Whitefly classification	Thrips classification
LA2172	*S. arcanum*	susceptible	resistant
LA1401^a^	*S. cheesmaniae*	susceptible	resistant
LA1840	*S. chmielewskii*	susceptible	susceptible
LA2695	*S. chmielewskii*	resistant	resistant
LA0407	*S. habrochaites*	susceptible	resistant
LA1777	*S. habrochaites*	resistant	resistant
PI134418	*S. habrochaites*	resistant	resistant
LYC4	*S. habrochaites*	resistant	resistant
LA1718	*S. habrochaites*	resistant	susceptible
PI127826	*S. habrochaites*	resistant	susceptible
LA1364	*S. huaylasense*	susceptible	susceptible
Moneymaker	*S. lycopersicum*	susceptible	susceptible
LA4024	*S. lycopersicum*	susceptible	susceptible
LA2133	*S. neorickii*	susceptible	susceptible
LA0735	*S. neorickii*	susceptible	susceptible
LA0716	*S. pennellii*	resistant	resistant
LA1278	*S. peruvianum*	susceptible	resistant
LA1954	*S. peruvianum*	resistant	susceptible
LA1578	*S. pimpinellifolium*	susceptible	susceptible

Regression analysis on the no-choice survival data with generalised linear model for whitefly data; *p* < 0.05 and Cox proportional hazards coefficients for thrips; *p* = 0.01, divides the tomato accessions in either “resistant” or “susceptible” environments for the insects. ^a^ LA1401 morphotype *S. cheesmaniae* [25, 53].

**Table 2:** Significant metabolites according to the random forest models

Metabolite	m/z	Experimental KI	Theoretical KI	Insect	Classification	***P***-value
S3:15	617 [M + Na]^+^	–	–	whitefly	resistance	*p* < 0.001
S3:21	715 [M + Na]^+^	–	–	whitefly	resistance	*p* < 0.001
α-humulene	204.2	1457	1454	whitefly	susceptibility	*p* < 0.001
α-phellandrene^a^	136.1	1000	1002	thrips	susceptibility	*p* < 0.001
α-terpinene^a^	136.1	1013	1017	thrips	susceptibility	*p* < 0.001
β-phellandrene/D-limonene	136.1	1027	1029	thrips	susceptibility	*p* < 0.001

Acylsugars are annotated by their sucrose (S) or glucose (G) backbone followed by the number of acyl chains and the number of carbon atoms distributed over the acyl chains. Volatiles were annotated using the Kovats Retention Index (KI) and fragmentation-pattern comparison to the Adams [46] and NIST libraries. The table provides the resistance/susceptibility classification for the different insects by the RF model and the *p*-value indicates whether the calculated feature importance significantly deviates from a randomly permuted model (threshold: *p* < 0.05). ^a^ Metabolite annotation confirmed by an analytical standard. The β-phellandrene/D-limonene peak could not be separated due to their strong co-elution. Mass spectra of metabolites not verified by an analytical standard are given in Additional file: Figure S8.

The RF model next linking volatiles to whitely resistance classifications had an accuracy score of 63% and indicated only one out of the 86 volatiles as a feature of significant importance (*p* < 0.01; Additional file: Figure S6c). The significant sesquiterpene, α-humulene (Table 2) was prevalent in whitefly-susceptible accessions (Fig. 5c).

### Linking specialised metabolites to thrips resistance

As noted earlier, thrips survival on the different genotypes differed from that of whitefly. Therefore, the accessions were again classified into two distinct groups, i.e. resistant-or susceptible to thrips (Table 1) after which the importance of individual features was computed. The thrips-acylsugar RF model exhibited a relatively low accuracy score of 58% and did not predict any acylsugar with a feature importance that significantly deviated from the randomly permuted models. The model based on volatile data exhibited a better accuracy score of 85% and resulted in three significant volatile peaks, all monoterpenes, including α-phellandrene, α-terpinene and β-phellandrene/D-limonene (Table 2; Additional file: Figure S7 and Additional file: Figure S8). Plotting their abundances showed that the monoterpenes α-phellandrene and α-terpinene were both predominantly present in thrips susceptible plants (Fig. 5d-e). The other volatile peak, β-phellandrene/D-limonene also occurred in some of the thrips resistant accessions (Fig. 5f).

## Discussion

Wild tomatoes can produce specific metabolic compounds that improve resistance to a variety of insects [24, 25, 54–57]. Plant resistance is generally a broadly used term, first delineated by Painter in 1951 [14] and could be described as “a heritable trait that minimises the damage experienced by the plant” [15]. Insect response to a plant can be recorded in numerous ways and the choice of measure can greatly impact the conclusions drawn [15]. Here we investigated a short-term antibiosis response for whitefly and thrips providing a measure of direct constitutive defence. The level and specificity of such resistance varies greatly among the selected accessions (Fig. 1). Our work corroborates that plant resistance properties against herbivorous insects cannot be generalised, but rather are defined by a specific (biochemical) environment in combination with the insect [25, 29, 56]. Indeed, this does not only hold true for the insect species but also for their life stages and it depends on the parameters chosen e.g. development, oviposition, survival, repellence [15]. Considering the resistance levels observed here, it suggests that tomato accessions harbour particular defence metabolites targeting adult whiteflies and thrips larvae. Although the mode of action of specialised metabolites on insects is not always fully understood, effects on cellular levels including respiration, membrane integrity, cellular transport and receptors have been described [21, 58, 59]. By choosing for survival assays, alternative defence mechanisms acting through repellence or distorting development were not included here. Hence wild-tomato metabolites we identified earlier as repellents, i.e. the volatile terpenes 7-*epi*-zingiberene and *R*-curcumene from PI127826, would not be discerned here to be associated to survival as they turned out not to be toxic [24, 60]. In addition, there are defence metabolites that are not produced by trichomes as well as enzymes and cuticle structures that could be involved in insect survival [61–63]. The choice for trichome-produced defence metabolites is however interesting in light of breeding for plant-produced specialised metabolites, as an alternative for the application of synthetic pesticides. Tomato cultivars can develop different types of trichomes, including type IV and VI on the green tissues, and while trichomes can store high levels of (auto)toxic metabolites, they are barely present on the mature tomato fruit [64]. The introgression of trichome-specific traits from wild species to cultivars, especially a combination of repellent and toxic metabolites, might therefore be a feasible option to improve resistance. The complexity of metabolite mixtures found trichomes often makes it hard to determine the effective individuals and we therefore aimed for a strategy that included metabolite types (i.e. acylsugars and volatiles) that have been implicated in resistance before.

The general relationship between (glandular) trichome density and the impact on herbivores is well-described in tomato [47, 65–67]. This is not always a negative relation though. In fact, whiteflies prefer to oviposit on hirsute leaves as - especially non-glandular - trichomes offer protection from natural enemies, such as parasitoids, to the nymphs [68–70]. Here we found indications that the impact of trichomes on insects appears attributable to its chemical content and that glandular trichome density is not necessarily a reliable indicator of resistance (Fig. 2). Type-VI trichome density did not correlate with the survival rates of either whitefly or thrips and the results imply that for this set of tomatoes type-VI trichome-related resistance depends more on the nature of metabolites than on the dose. Indeed, insects can be resilient to plants producing a relatively high quantity of terpenoids (Additional file: Figure S5). However, we did observe a clear negative correlation between type-I/IV trichome density and adult whitefly survival (Fig. 2) in line with previous findings [28, 71, 72], indicating there is probably a dose-dependent effect of acylsugars on whitefly mortality. The resistant tomato accessions in the panel do indeed exhibit much higher acylsugar levels (Additional file: Figure S4). Recently it was indicated that the effect of acylsugar exudates on insect resistance depends on the type of sugar backbone and the composition of the acyl chains [28, 29, 59]. Leckie et al. [36] concluded that acylsugar exudates enriched in acylsucroses and 3-methylbutanoic (iC5) fatty acids would be most effective in reducing whitefly oviposition. We also found the majority of the resistant accessions to accumulate mainly tri-acylsucroses (Additional file: Figure S4). The model did not significantly distinguish the resistant-and susceptible accessions based on the total amount of tri-acylsucroses or sucrose backbones (Additional file: Figure S4), but rather predicted S3:15 and S3:21 specifically to impair adult whitefly survival (Table 2). Both compounds are in cluster 2 of Fig. 3A, which comprises compounds predominantly produced in resistant accessions. A study on greenhouse whitefly (*Trialeurodes vaporariorum*) resistance by Vosman et al. [56] showed that the renowned whitefly-resistant tomato accession *S. galapagense* LA1401 also contained high levels of acylsucroses, in which several isoforms of S3:21 are abundant. Although we could not determine the exact acylsugar configuration here due to methodological constraints, we can deduce the composition of the acyl chains as these compounds were previously elucidated in tomato. Ghosh et al. (2014) reported that the S3:21 isomers in LA1777 all included an iC5, a 2-methylbutanoate (aiC5) and a 9-methyldecanoate (iC11) chain, hence S3:21 (5,5,11). S3:15 found in, amongst others, *S. habrochaites* LA1718 and LA0407 were determined to contain three 3-methylbutanoate groups hence S3:15 (5,5,5) [73]. Besides the validation of the individual compounds, it would be valuable to see if indeed the esterification of iC5 acyl chains is particularly responsible for the resistance effect as noted by Leckie et al. [36].

Survival of the thrips larvae on the other hand, did not appear to be linked to any of the acylsugars present in the tomatoes studied. Firstly, survival did not correlate to the density of type-I/IV trichomes to the extent seen for whiteflies (Fig. 2c). Secondly, the RF model did not indicate any of the detected acylsugars present in this panel to be involved in separating thrips-resistant from thrips-susceptible accession. However, based on *S. pennellii* (LA0716) introgression material, Ben Mahmoud et al. [37] and Leckie et al. [36] did find indications that both acylsugar dose and the fatty-acid composition affected thrips oviposition rates. The protective effect of acylsugars against thrips could thus very well depend on the thrips’ developmental stage. We used the thrips larvae in our bioassays, which are perhaps less likely to come in contact with the glandular head-cells, thereby receiving a lower dose of acylsugars compared to the bigger and much more mobile adults.

Although the evidence for the importance of specific acylsugars, or their structural features, in plant resistance starts to accumulate, there are also indications for synergistic effects of acylsugars mixtures. Hence, a specific combination of acylsugars can cause a stronger effect on the insect than the sum of individuals compounds [36, 37]. Ideally next, validation experiments with individual and purified acylsugars S3:15 (5,5,5) and S3:21 (5,5,11) would be performed. However, such efforts remain challenging. Isolation, or synthesis, of single acylsugars is extremely difficult as they are complex molecules, with a high molecular similarity, generally present in varying mixtures in plants (Fig. 3a) [74]. Alternatively, creating a segregating population from one of the resistant accessions identified here, or making use of tomato introgression lines with different acylsugar profiles such as done by, amongst others, Leckie et al. [74], Smeda et al. [75] and Ben-Mahmoud et al. [37], could help reveal a causal link between a specific metabolite (combination) and the effect on different types of pests.

The sesquiterpene α-humulene was the only volatile predicted to distinguish whitefly resistant from whitefly-susceptible accessions. This compound is present in susceptible lines and was previously reported to potentially serve as a host attraction cue [76, 77], a response not tested here. The effect observed appears to be compound specific though, as the total volatile quantity (based on peak area) or structural metabolic group, did not discriminate resistant from susceptible accessions after RF modelling (Additional file: Figure S5). The model predicted three monoterpene peaks, α-phellandrene, α-terpinene and β-phellandrene/D-limonene to separate thrips susceptible from resistant accessions. These compounds were particularly prevalent in susceptible accessions and do not affect thrips survival. However, upon activation of the Jasmonate signalling-pathway in *S. lycopersicum* cultivars, the abundance of monoterpenes, including the ones described above, increases resulting in a decreased preference response of adult thrips [78]. So, although our results suggest that these monoterpenes do not have a toxic effect on the thrips larvae, they could play a role in the repellence of the adults. Also, here short-term performance assays were done on naïve plants, not primed by earlier damage, focussing on constitutive and direct defence, but both thrips and whitefly infestations have been shown to induce specific chemical defence in accordance with their feeding style and can even manipulate host response in neighbouring plants [79, 80], highlighting the complexity of this dynamic trait.

While resistance against thrips is noticeably different between accessions (Fig. 1b; Additional file: Figure S2) this may involve trichome-independent metabolites such as (glyco)alkaloids, as was recently suggested for thrips feeding behaviour [81]. Alternatively, it might still be that volatile metabolites measured here, may be involved but were not selected because of sparsity in the dataset. This is supported by the observation that a relatively large portion of the metabolites were exclusively detected in a single accession (Fig. 3). The metabolic diversity in the set of tomatoes used here makes statistical analysis challenging, especially since the number of resistant accessions with overlapping metabolites is small, particularly in case of the volatiles. As noted above, it might mean that a uniquely appearing metabolite, despite having biological activity, was not selected only due to a lack of statistical power. For example, we recently identified the 7-*epi*-zingiberene derivative 9-hydroxy-10,11-epozyzingiberene (9H10epoZ) to be toxic to whiteflies [60]. This compound is produced by, and specific to, *S. habrochaites* PI127826 (Fig. 3b), and was therefore probably not marked as a significant discriminant by the RF model. Another example is santanaloic acid which, as mentioned before, is toxic to *H. zea* and *S. exigua* larvae [44] and potentially to other insects as well [82]. This metabolite was also not selected by our model, as it was exclusively detected in *S. habrochaites* accession LA1777. These examples indicate the limitations of a model when metabolic diversity is broad with too few overlapping accessions. The use of more closely related chemotypes (e.g. segregating populations or accessions of the same species) displaying contrasting resistance phenotypes would therefore be ideal for RF analyses.

Nevertheless, the RF method chosen here resulted in the selection of specialised metabolites from complex mixtures of acylsugars and volatiles in tomato glandular trichomes that could discriminate resistant from susceptible genotypes. Regression-based classification-methods like PLS-DA, widely applied in metabolomics and plant-insect interaction studies in particular [83–86], could not be applied here, as a consequence of the sparsity, or zero-valued elements, and the high feature to sample ratio [87, 88]. Even sparse PLS-DA [89] led to impaired model convergence. The RF approach is regarded to be a powerful classification method, especially in case of sparse data [50, 90] and a low sample-to-feature ratio, common in multi-omics analyses [91]. In addition, the comparison of the RF results to a permuted dataset allowed statistical discrimination of the importance of individual metabolites. Despite its promise only a few examples of the use of RF for metabolite selection in plant science are available, including metabolite selection for potato-tuber discolouration [92], fingerprinting of primary metabolism in *Arabidopsis* mutants [93] and sex-related volatiles emitted by the cones of *Ficus* species [94]. Here we pose that RF can predict specific metabolites with potential in defence from a challenging dataset, making it a promising tool in future identification of specialised metabolites with potential biological significance from complex mixtures often found in plants.

Breeding for metabolite-based resistance is challenging and requires the introgression of several wild-species QTLs to obtain effective concentrations of herbivore-specific metabolites. Advances towards insect resistance in tomato have been made by creating pre-breeding material, introgression lines containing metabolite-QTLs that can be combined for insect-specific resistance, especially for acylsugars [28, 30, 36, 37, 74, 95]. The approach presented here can be complementary. The prediction of specific bioactive compounds that are causal to the desired phenotype enables a more targeted approach for elucidation of the genetic components underlying the metabolite composition, and a more efficient incorporation of insect resistance into breeding material [31, 96–98]. For agriculture, losses caused by herbivorous insects can be largely attributed to their vectoring of viruses. Therefore, treatment of the vector insects will impact also the pathogens they carry. The two candidate acylsugars we predicted to negatively affect whitefly survival are expected to additionally decrease the efficient spread of virus by this vector [30].

## Conclusions

While there are many reports on the insecticidal properties of essential oils and plant extracts, there is only limited information about individual biologically active compounds [99]. Here we saw that diverse accessions that vary in metabolite profile can have different effects on different vector insects, and we put forward a set of specialised metabolites with the potential to act as natural insecticides. In the material analysed two specific acylsugars appear to have potential for chemical defence against whiteflies, however not for thrips larvae. Breeding for metabolite-based resistance poses a challenge that requires a clear definition of the desired trait and incorporation of multiple QTLs. By the use of RF we were able to assign specific specialised metabolites from complex mixes to distinct phenotypic traits. The model can be improved further using accessions that display more overlap in metabolic profiles, such as in more closely related species or in segregating populations with distinct resistance profiles.

## Methods

### Plant and insect materials

The panel of tomato accessions was aimed to be diverse in specialised-metabolite profiles and contrasting in insect resistance. Based on previous SNP annotations [100] and in combination with seed availability we selected 19 accessions from 10 different tomato species. A relatively large number of accessions belong to the species *Solanum habrochaites* (previous known as *S. typicum f. glabratum* or *S. typicum f. hirsutum*), as these are well known for herbivore resistance and specialised metabolite production [22–24, 27, 54, 73, 101–106]. Tomato accessions were obtained from Enza Zaden, Enkhuizen according to the appropriate guidelines and licences for plant material. Plants were grown in an enclosed greenhouse compartment under controlled conditions (22–25 °C, 16/8 h light/dark regime). All leaf material used in the experiments originates from leaflets of similar developmental stage and plant age. Unless otherwise indicated, the first pair of leaflets from the fourth fully expanded leaf from the apex of a whole plant was used. For each experiment (i.e. whitefly bioassay, thrips bioassay, trichome density, acylsugar measurements, volatile measurements), a separate batch of plants was grown from seed while randomly distributed over the same greenhouse compartment.

The *B. tabaci* population (MEAM1) was reared in a climate cabinet (Snijders, Tilburg; 16/8 h light/dark; 150 μE m^− 2^ s^− 1^ at 28 °C) on fresh cucumber plants. The Western Flower Thrips (*F. occidentalis*) population was reared on runner bean supplemented with *Typha latifolia* pollen in an ECO2box plastic container (Duchefa Biochemie, Haarlem, The Netherlands), located in a climate cabinet (16/8 h light/dark; 150 μE m^− 2^ s^− 1^ at 24–20 °C).

### Insect bioassays and statistics

Bioassays were tailored to the specific insect to provide the most reliable read out. The resistance of 19 different tomato accessions to whitefly (*B. tabaci*; MEAM1) were determined using a no-choice bioassay to obtain the mean percentage of whitefly survival. Whitefly bioassays were performed using 6-weeks old plants randomly localised over an enclosed greenhouse compartment (22–25 °C; 16/8 h light/dark). Per tomato accession, 3 individual plants were used except for PI127826 (*n* = 5) and MM (*n* = 8) used to monitor block- and positional effects. Each plant harboured two clip cages (ø 2.5 cm, Bioquip, cat #1458) placed on the fourth fully expanded leaf from the apex. The cages were filled with ~ 15 adult whiteflies and after 5 days the number of dead and alive whiteflies per clip cage was recorded using a stereomicroscope (Euromex StereoBlue Zoom SB.1902). Whitefly survival (%) was first averaged over the two clip cages to obtain an average survival per plant before calculating the accession’s average survival and standard error. To create two phenotypic groups as input for the RF modelling, a generalised linear model was fitted using the *glm* and the *glmer* function of R package *lme4* [107, 108] taking the variable “accession” as a fixed effect and using *S. lycopersicum* accession Moneymaker, functioning as a sensitive standard, as the intercept. Whitefly survival data from *S. pennellii* LA0716 was excluded from this analysis; as the survival was 0%, the coefficient could not be calculated. Accessions with statistically significant (*p* < 0.05) coefficients were labelled as “resistant” and others as “susceptible”.

For the thrips assays we assayed larval performance, as thrips become virus vectors at this life stage, hence establishing the median survival time as survival parameter. For this, ø 15 mm leaf discs were placed in a 12-well plate (Greiner-bio one #665102) on top of a ø 20 mm filter paper (Whatman #28413904) with 100 μl of tap water to keep the leaf discs hydrated. Per well (24 to 36 wells per accession) one thrips larva (L1 stage) from a synchronised population was placed on the adaxial leaf surface of a leaf disc. Plates were closed, sealed with tape and placed back into the climate cabinet after which the survival status, 1 (dead) or 0 (alive), of each individual was documented daily for 19 days. Kaplan-Meier survival curves (Additional file: Figure S1) were drawn in R [108] using the *survfit* function [109] and the *ggsurvplo*t function of the *survminer* package version 0.4.0 [110]. A Cox proportional hazard model, as previously used by Macel et al. [111], was fitted using the *coxph* function of the *survival* [109] package with the accession as explanatory variable (Additional file: Table S2). For each tomato accession, the fitted Cox proportional hazard regression model estimated the probability coefficient for a thrips L1 larva to die when exposed to a leaf disc, compared to the baseline (susceptible standard *S. lycopersicum* Moneymaker). For instance, *S. habrochaites* LYC4 has an exponentiated coefficient of 215 meaning that the chance of dying on a LYC4 leaf disc is 215 times greater compared to MM. Accessions with a statistically significant (*p* < 0.01) coefficient higher than 1 were labelled as “resistant” and otherwise as “susceptible”.

### Trichome density and microscopy images

Leaflets were taken from 3 to 5 individual plants per accessions, from which four leaf discs (ø 4 mm) were taken; two leaf discs were used to count trichomes on the abaxial side and two for the adaxial side. For all, type II/III/V/VIII (“non-glandular”), glandular type I/IV and type VI were counted using a stereomicroscope (Euromex StereoBlue Zoom SB.1902). Type I and IV were taken together as one group as the two types are related and contain similar acylsugar compositions in the glands. Moreover, though type I should have a taller stalk than type IV [112] this criterium was impossible to distinguish varying in the wild accessions. In the absence of a statistically significant leaf disc effect (*p* > 0.05), trichome density (trichomes/mm^2^) was averaged over the two leaf discs from an individual leaflet (plotted in Additional file: Figure S3), whereafter the average trichome density per accession was calculated. As the setup of the insect-bioassay allowed whiteflies to move freely between both sides of the leaflet, trichome densities were averaged over the abaxial-and adaxial surface to analyse the effect of trichome density on whitefly survival (Fig. 2b; Additional file: Figure S3c). Thrips larvae were restricted to reside only on the adaxial surface and the survival rates were therefore compared to trichome densities of the adaxial surface only (Fig. 2c; Additional file: Figure S3d). Linear modelling of the trichome densities and insect-survival rates was done in R using the simple linear modelling *lm* function.

Pictures of wild- and cultivated tomato trichomes were obtained using a Leica MZFLIII stereomicroscope connected to a Nikon DS-Fi2 digital camera. Tissue was placed under the microscope and photos were taken in layers of ~ 10 nm under bright light. Layers (20 to 60 photos) were stacked into a single image using layer stacking in Adobe Photoshop (CC 2019, Adobe Systems).

### Metabolic profiling

#### Metabolite extractions

Volatiles were extracted from one leaflet (*n* = 3–4), that was weighed and briefly (~ 5 s) immerged in 500 μL *n*-hexane spiked with 0.5 ng/μL benzyl acetate as internal standard. The short extraction time served to avoid extraction additional cuticle components, which was subsequently monitored. Next, 10 mg Na_2_SO_4_ (s) was added to the extract to remove water and the sample was vortexed for ~ 10 s. Extracts were then centrifuged for 5 min at 13.9 k rcf whereafter the hexane layer was removed and stored in glass vials under N_2_ (g) at − 20 °C prior to the analysis. For acylsugar extractions, two leaflets (*n* = 6) were collected, weighed and immerged in 2 mL dichloromethane while gentle rocking for 30 s. The extracts were air-dried overnight, stored at − 20 °C, and dissolved in 300 μL MeOH for analysis.

#### Data acquisition and identification

Volatile analysis was done using an Agilent 7890A gas chromatograph coupled to a 7200 accurate mass time-of-flight (TOF) mass spectrometer. One μL of extract was injected, immediately heated to 275 °C and separated on a HP-5 ms column (30 m × 250 μm; 0.25 μm film thickness; Agilent) using helium as a carrier gas (7.0699 psi; flow rate: 1 mL/min). The column was heated for 3 min at 40 °C thereafter the temperature increased with 5 °C/min to 140 °C, then 10 °C/min to 250 °C which was held for 5 min. Samples were ionised at 70 eV under vacuum using EI. After a solvent delay of 4.1 min, ions in the range of 30–350 mu were detected at 50 scans/second. Peak detection was done using MassHunter Qualitative Analysis software package (Agilent). After chromatogram deconvolution, peaks were picked with 50 ppm accuracy when having a minimum ion count of 1% compared to the highest peak in the chromatogram and a S/N ratio > 10. Metabolite identification was done by mass spectrum comparison to the NIST- and Adams (2007) libraries [113, 114] in combination with the Kovats Retention Index (KI) [115], calibrated by running a C8-C20 alkane standard (Sigma Aldrich #04070-5ML). Identifications were verified by analytical standards when available and peaks with a library hit of low confidence were labelled as “unknown” (Additional File: Table S3). Due to their strong co-elution β-phellandrene and D-limonene peaks could not be separated and were treated as one. Metabolite peaks were integrated by their base-peak ion and peak areas were corrected by the internal standard and sample dilution and normalised by the fresh-leaf weight.

Acylsugar extracts were analysed using an Agilent 1290 Infinity II UHPLC coupled to an Agilent 6230 TOF mass spectrometer. One μL of extract was injected and separated on an Agilent ZORBAX RRHD Eclipse Plus C18 column at 50 °C with a mobile-phase flow-rate of 0.3 mL/min. The mobile phase consisted of water + 0.1% formic acid (A) and acetonitrile + 0.1% formic acid (B) in the following A:B gradient; from 60:40 to 45:55 in 6 min to 10:90 in 8 min to 60:40 in 3 min. Molecules were ionised at 325 eV in positive mode and ions were detected in the range of 50–1500 mu at 1 spectrum/sec. Acylsugars were identified using MassHunter Quantitative Analysis software (Agilent) by calculating the molecular formulas of the parent ion constituting the chromatographic peaks. Here, molecular formulas were constrained by allowing carbon, hydrogen and oxygen atoms to reconstruct the parent ion, in combination with H^+^, Na^+^ and K^+^ and formate adducts to appear, plus a double bond equivalent (DBE) range of 1–10. The generated molecular formula, in combination with the DBE, allows extrapolation of the acylsugar molecular structure; the backbone moiety, number of esterified acyl chains and the total number of carbon atoms constituting the acyl chains. Structural isomers were labelled by a hyphen followed by a number.

### Random forest

As input for the RF classification algorithm, metabolite levels of the accessions were averaged across biological replicates and the accessions’ classification (i.e. “resistant” or “susceptible”) was taken from the regression analysis. Every RF model was run by a six-fold cross validation scheme to build training-and test sets using 1000 decision trees, yielding a feature importance for every metabolite. The model was run 5 times with random initialisation parameters producing an average feature importance and standard deviation for each metabolite (Fig. 4, step 1). Next, the significance of each metabolite’s average feature importance was evaluated by comparison to feature-importance distributions obtained from 100, 250 and 500 RF models using randomly-permutated datasets (Fig. 4, step 2). A metabolite was regarded significantly contributing to phenotypic classification when the calculated average feature importance was higher than in 95% of the 100 permuted RF models (*p* < 0.01; Fig. 4, step 3). The modelling was performed using the Python Machine Learning library *scikit-learn* implementation [116] in the Python Pandas data structure [117]. The full script is available as Jupyter notebook in the companion GitHub repository (http://www.github.com/BleekerLab).

Methods and plant material described above complied with relevant institutional, national and international guidelines and legislation.

## Supplementary Information

**Additional file 1: Figure S1.** Kaplan-Meier survival curves of the thrips bioassay. The data shows the survival of the thrips larvae over time on leaf discs of the accessions used in this study.

**Additional file 2: Figure S2.** Trichome photos of all 19 accessions. The photos illustrate the diverse trichome landscape that can be found over the accessions.

**Additional file 3: Figure S3.** Trichome densities per trichome-type and leaf surface. Boxplots showing the individual data points used to calculate the average trichome densities and the scatterplots show the relationship between trichomes densities and insect survival rates that were not displayed in the main figures.

**Additional file 4: Figure S4.** Overview of the acylsugar composition of the accessions. The plots display the contribution of the different structural features of the detected acylsugars measured in each accession and the relationship between the total ion-counts and insect survival rates.

**Additional file 5: Figure S5.** Overview of the structural composition of volatile metabolites. The plots display the contribution of each structural class to the total blend of volatiles measured in each accession and the relation between the total ion-counts and insect survival.

**Additional file 6: Figure S6.** Feature importance of metabolites as computed by the whitefly-metabolite RF-models versus their distribution in permuted models. Only metabolites with a feature importance significantly higher compared to the permuted models are shown.

**Additional file 7: Figure S7.** Feature importance of metabolites as computed by the thrips-volatiles RF-models versus their distribution in permuted models. Only metabolites with a feature importance significantly higher compared to the permuted models are shown.

**Additional file 8: Figure S8.** Mass spectra of metabolites with a significant feature importance that could not be verified by an authentic standard. Given spectra were used for identification by library comparison in combination with their Kovats Retention Index.

**Additional file 9: Table S1.** Generalised Linear Model fitted on the whitefly survival data. The output of the model was used to classify accessions as either resistant or susceptible to whiteflies which served as an input for the random forest algorithm.

**Additional file 10: Table S2.** Cox proportional hazard model fitted on the thrips survival data. The output of the model was used to classify accessions as either resistant or susceptible to whiteflies which served as an input for the random forest algorithm.

**Additional file 11: Table S3.** Normalised peak-area of the volatiles and acylsugars detected over the samples. The file contains the data that was used as input material for Fig. 3, Fig. 5 and the random forest models and includes the experimental-and theoretical Kovats Retention Indices used for identification of the volatiles.

## Data Availability

All data generated during this study are included in this published article and its supplementary information files. We provide the underlying datasets, and code as R-scripts and Jupyter notebooks, through a linked GitHub repository (https://github.com/BleekerLab/natural-insecticides-thrips-whiteflies) and at the Zenodo archive (https://zenodo.org/).

## Abbreviations

DBE: Double bond equivalent
GC-MS: Gas chromatography-mass spectrometry
KI: Kovats retention index
MM: MoneyMaker
NG: Non glandular
PLS: Partial least squares
PLS-DA: Partial least squares discriminant analysis
Q-TOF: Quadrupole time-of-flight
RF: Random forest
UHPLC: Ultra high-performance liquid chromatography
iC5: 3-methyl-butanoate
aiC5: 2-methyl-butanoate
iC11: 9-methyl-decanoate
