# Supplementary material for: Natural variation in wild tomato trichomes; selecting metabolites that contribute to insect resistance using a random forest approach
Source: BMC Plant Biol. 2021 Jul 2;21:315. doi: 10.1186/s12870-021-03070-x (PMC8252294; doi:10.1186/s12870-021-03070-x)
Supplement: Supplementary file 1 — Additional file 1: Figure S1. Kaplan-Meier survival curves of the thrips bioassay. The data shows the survival of the thrips larvae over time on leaf discs of the accessions used in this study. [file 12870_2021_3070_MOESM1_ESM.pdf]

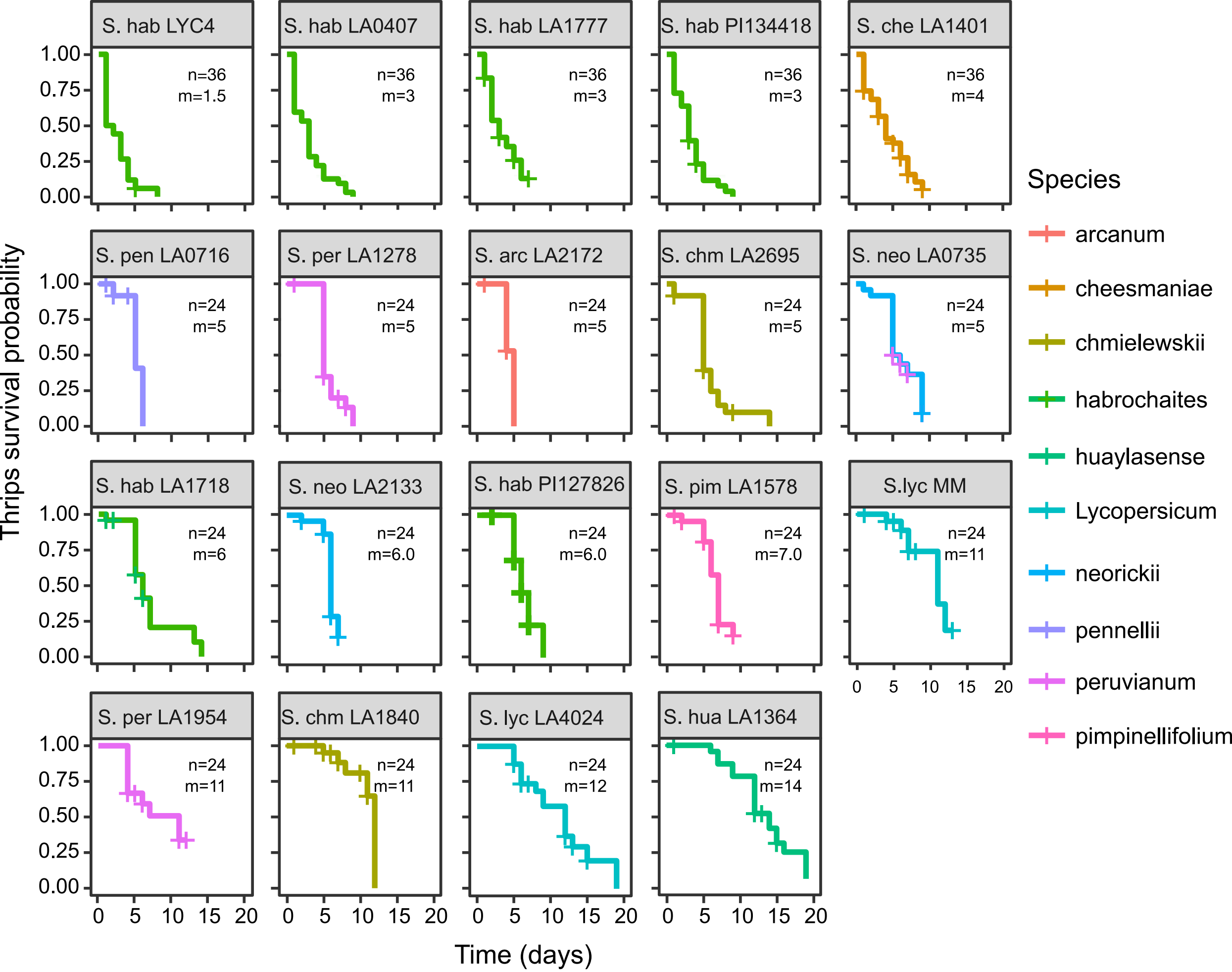

Supplementary Fig. S1. Western flower thrips Kaplan-Meier survival curves on the different accessions used to calculate the median survival time in **Fig. 1B**. Western flower thrips survival over time (19 days) was estimated using the Kaplan-Meier estimation method. One L1 larvae was placed on a 1.5 cm ø leaf disc (n = 24-36) and survival was recorded daily. Tomato genotypes are ordered by the median survival time (i.e. the day that 50% of the larvae died) also indicated in the graph (m). Crosses indicate censored observations i.e. thrips that were missing at the time of measurement or that survived until the end of the experiment. The 95% confidence interval is indicated with the lighter colour.
