# Supplementary material for: Natural variation in wild tomato trichomes; selecting metabolites that contribute to insect resistance using a random forest approach
Source: BMC Plant Biol. 2021 Jul 2;21:315. doi: 10.1186/s12870-021-03070-x (PMC8252294; doi:10.1186/s12870-021-03070-x)
Supplement: Supplementary file 2 — Additional file 2: Figure S2. Trichome photos of all 19 accessions. The photos illustrate the diverse trichome landscape that can be found over the accessions. [file 12870_2021_3070_MOESM2_ESM.pdf]

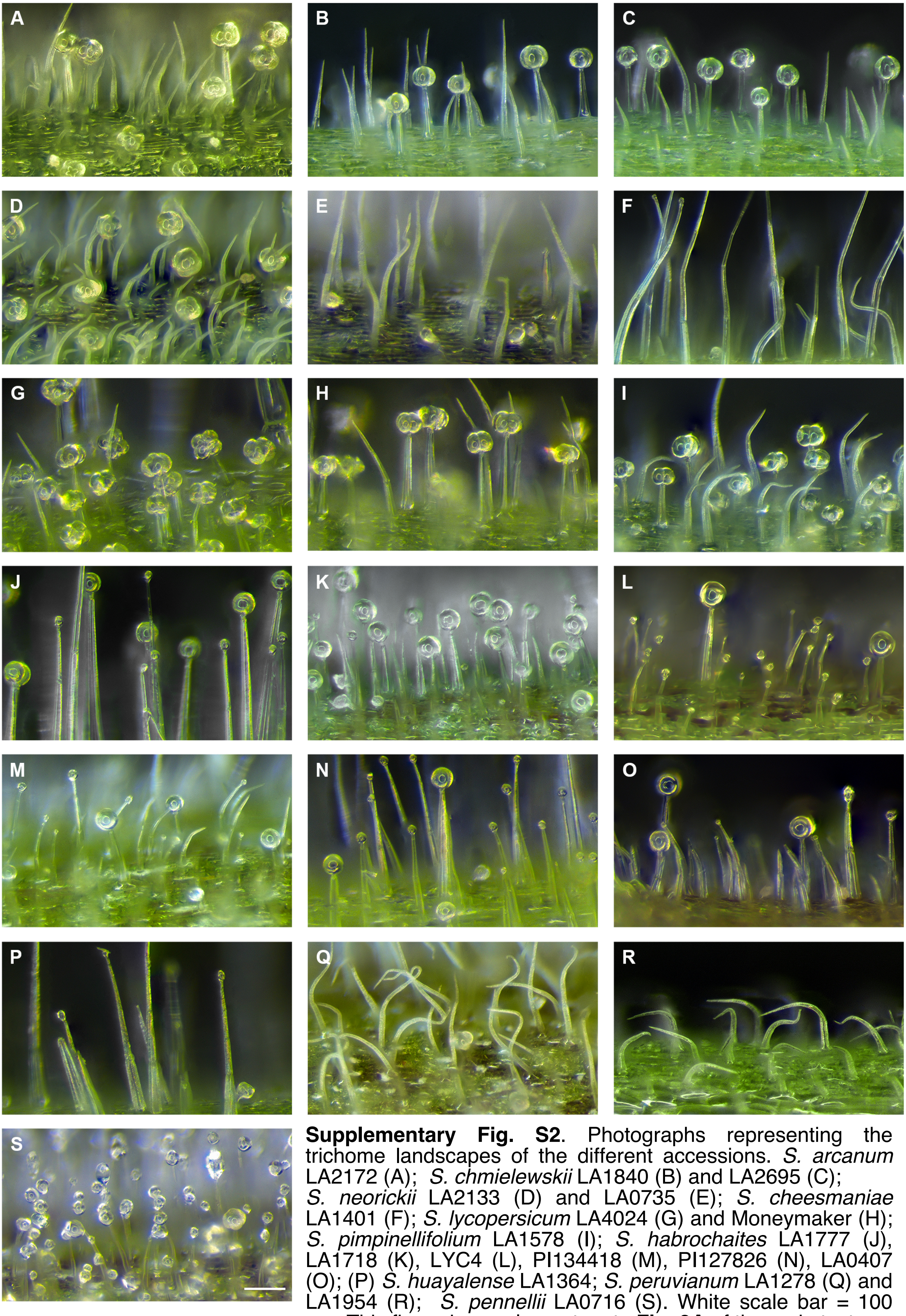

Supplementary Fig. S2. Photographs representing the trichome landscapes of the different accessions. *S. arcanum* LA2172 (A); *S. chmielewskii* LA1840 (B) and LA2695 (C); *S. neorickii* LA2133 (D) and LA0735 (E); *S. cheesmaniae* LA1401 (F); *S. lycopersicum* LA4024 (G) and Moneymaker (H); *S. pimpinellifolium* LA1578 (I); *S. habrochaites* LA1777 (J), LA1718 (K), LYC4 (L), PI134418 (M), PI127826 (N), LA0407 (O); (P) *S. huayalense* LA1364; *S. peruvianum* LA1278 (Q) and LA1954 (R); *S. pennellii* LA0716 (S). White scale bar = 100 μm. This figure is supplementary to **Fig. 2A** of the main text.
