# Supplementary material for: Natural variation in wild tomato trichomes; selecting metabolites that contribute to insect resistance using a random forest approach
Source: BMC Plant Biol. 2021 Jul 2;21:315. doi: 10.1186/s12870-021-03070-x (PMC8252294; doi:10.1186/s12870-021-03070-x)
Supplement: Supplementary file 4 — Additional file 4: Figure S4. Overview of the acylsugar composition of the accessions. The plots display the contribution of the different structural features of the detected acylsugars measured in each accession and the relationship between the total ion-counts and insect survival rates. [file 12870_2021_3070_MOESM4_ESM.pdf]

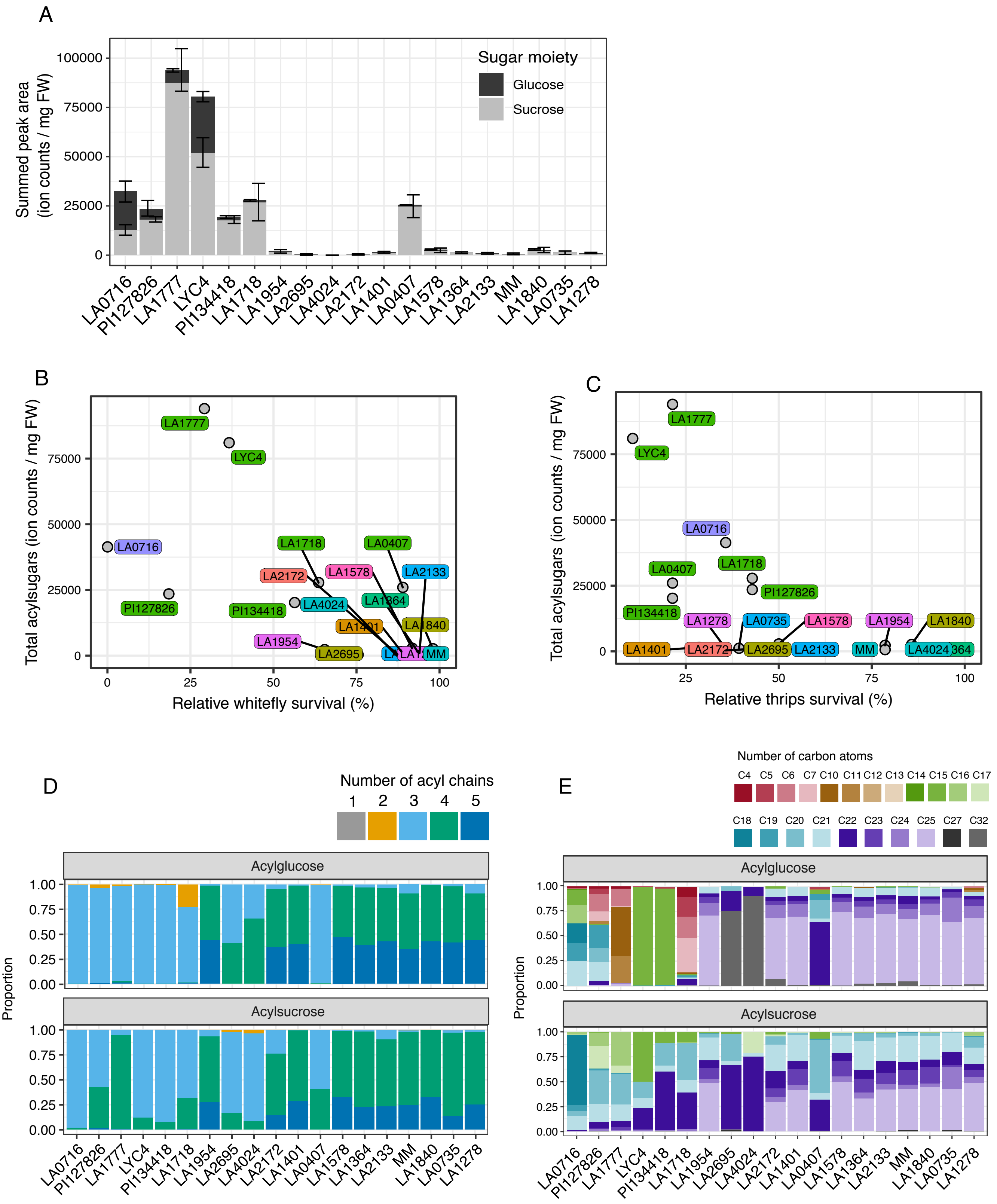

Additional file: Figure S4. Overview of the acylsugar compositions of the accessions. The accessions are ordered from low to high whitefly survival rate. **(A)** Summed peak area of the detected acylsucroses (light-grey bars) and acylglucoses (dark-grey bars). The bars indicate the mean peak area over 6 biological replicates ± SE. (**B**) Averaged summed peak area of all acylsugar plotted against whitefly survival rates. (**C**) Averaged summed peak area plotted against thrips survival rates. **(D)** Proportion of acylsugars with *n*-acyl chains esterfied to their sugar backbone for the detected acylglucoses (top panel) and acylsucroses (lower panel). Proportions are based on the mean peak area of the mother ions over 6 biological replicates. **(E)** Proportion of the total number of carbon atoms that constitute the acylchains in the pool of acylglucoses (top panel) or acylsucroses (lower panel) based on the mean peak area of the mother ions of the 6 biological replicates. This figure is supplemental to Figure 2 sof the main text.
