# Supplementary material for: Natural variation in wild tomato trichomes; selecting metabolites that contribute to insect resistance using a random forest approach
Source: BMC Plant Biol. 2021 Jul 2;21:315. doi: 10.1186/s12870-021-03070-x (PMC8252294; doi:10.1186/s12870-021-03070-x)
Supplement: Supplementary file 5 — Additional file 5: Figure S5. Overview of the structural composition of volatile metabolites. The plots display the contribution of each structural class to the total blend of volatiles measured in each accession and the relation between the total ion-counts and insect survival. [file 12870_2021_3070_MOESM5_ESM.pdf]

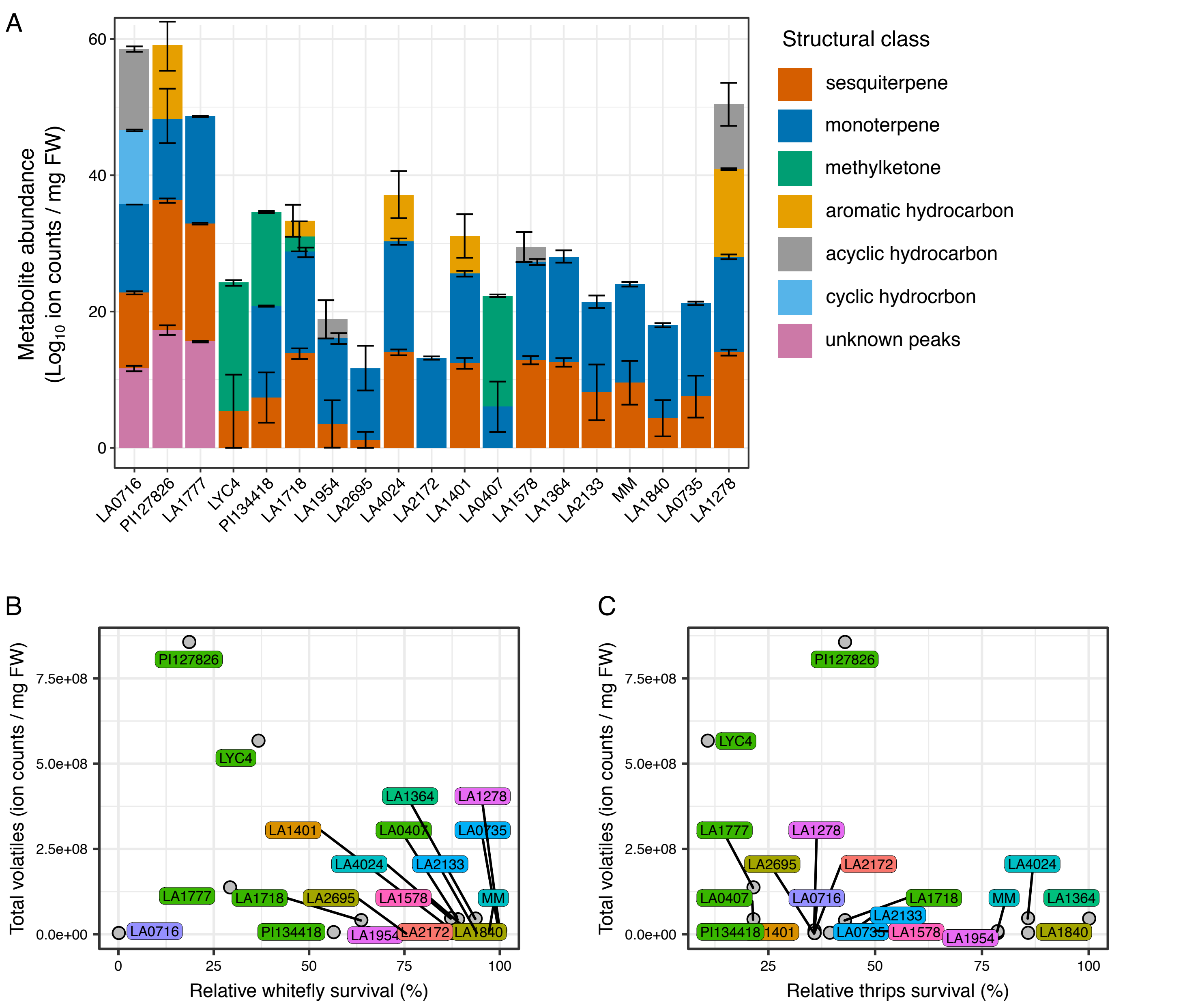

Additional file 5: Figure S5. Summed peak areas of the volatile metabolites as detected by GC/MS-TOF. **(A)** Summed peak area of the different structural classes detect over the accessions. The accessions are ordered from a low to high whitefly survival rate. Bars represent the $\log_{10}$-transformed mean peak-area of the summed metabolites (quantified by the base-peak of each mass spectrum) categorised to the same structural class ± SE over 3-8 biological replicates. Metabolites that could not be matched to an entry in the avaialbe libraries are categorised as "unknown peaks". More details about the metabolite identification and categorisation can be found in Additional file: Table S3. **(B)** The averaged total ion counts plotted against whitefly survival rates. (**C**) The averaged total ion counts plotted against thrips survival rates. The figure is supplemental to Figure 3B of the main text.
