# Supplementary material for: Natural variation in wild tomato trichomes; selecting metabolites that contribute to insect resistance using a random forest approach
Source: BMC Plant Biol. 2021 Jul 2;21:315. doi: 10.1186/s12870-021-03070-x (PMC8252294; doi:10.1186/s12870-021-03070-x)
Supplement: Supplementary file 6 — Additional file 6: Figure S6. Feature importance of metabolites as computed by the whitefly-metabolite RF-models versus their distribution in permuted models. Only metabolites with a feature importance significantly higher compared to the permuted models are shown. [file 12870_2021_3070_MOESM6_ESM.pdf]

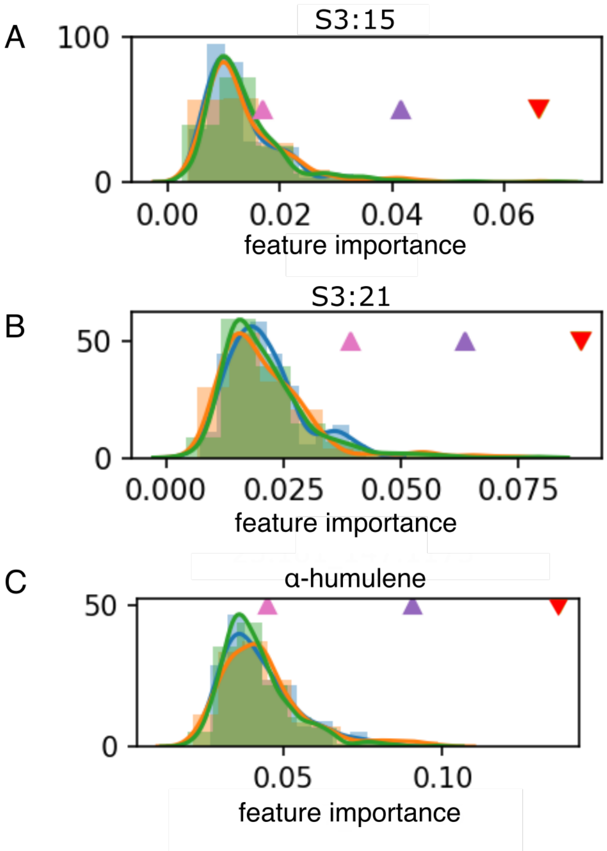

Additional file: Figure S6. Average feature importances from whitey-metabolite random forest runs related to the results of randomly permuted models. Distributions of metabolite feature importance's from 100, 250 and 500 permuted models are plotted in blue, orange and green respectively. The average feature importance over 5 random forest models is indicated with the purple triangle.
Pink triangle: average feature importance minus 2*Standard Deviation. Red trinangle: average feature importance plus 2*Standard Deviation. Shown are the acylsugars **(A, B)** and volatiles **(C)** with an average feature importance significantly deviation from their permuted models.
