# Supplementary material for: Natural variation in wild tomato trichomes; selecting metabolites that contribute to insect resistance using a random forest approach
Source: BMC Plant Biol. 2021 Jul 2;21:315. doi: 10.1186/s12870-021-03070-x (PMC8252294; doi:10.1186/s12870-021-03070-x)
Supplement: Supplementary file 7 — Additional file 7: Figure S7. Feature importance of metabolites as computed by the thrips-volatiles RF-models versus their distribution in permuted models. Only metabolites with a feature importance significantly higher compared to the permuted models are shown. [file 12870_2021_3070_MOESM7_ESM.pdf]

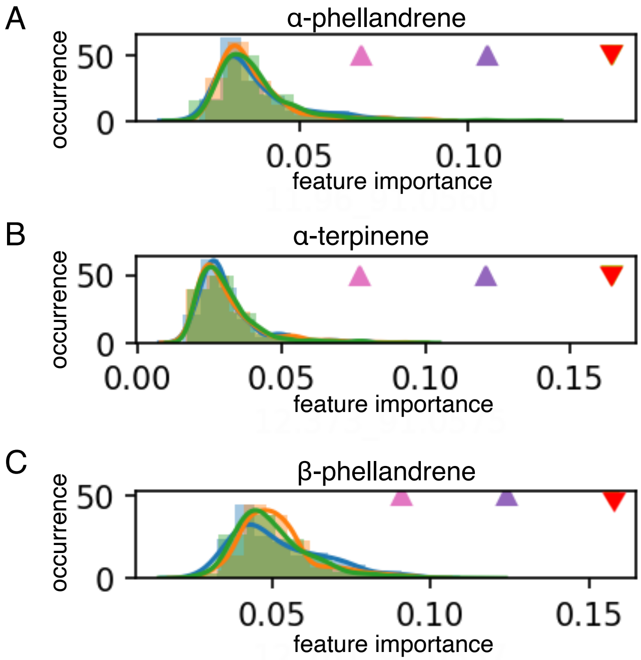

Additional file: Figure S7. Average feature importances from thrips-metabolite random forest runs related to the results of randomly permuted models. Distributions of metabolite feature importances from 100, 250 and 500 permuted models are plotted in blue, orange and green respectively. The average feature importance from 5 random forest models is indicated with the purple triangle.

Pink triangle: average feature importance minus 2*Standard Deviation. Red trinangle:average feature importance plus 2*Standard Deviation. **(A-C)** Volatiles with an average feature importance significantly deviating from their permuted models.
