# Supplementary material for: Natural variation in wild tomato trichomes; selecting metabolites that contribute to insect resistance using a random forest approach
Source: BMC Plant Biol. 2021 Jul 2;21:315. doi: 10.1186/s12870-021-03070-x (PMC8252294; doi:10.1186/s12870-021-03070-x)
Supplement: Supplementary file 3 — Additional file 3: Figure S3. Trichome densities per trichome-type and leaf surface. Boxplots showing the individual data points used to calculate the average trichome densities and the scatterplots show the relationship between trichomes densities and insect survival rates that were not displayed in the main figures. [file 12870_2021_3070_MOESM3_ESM.pdf]

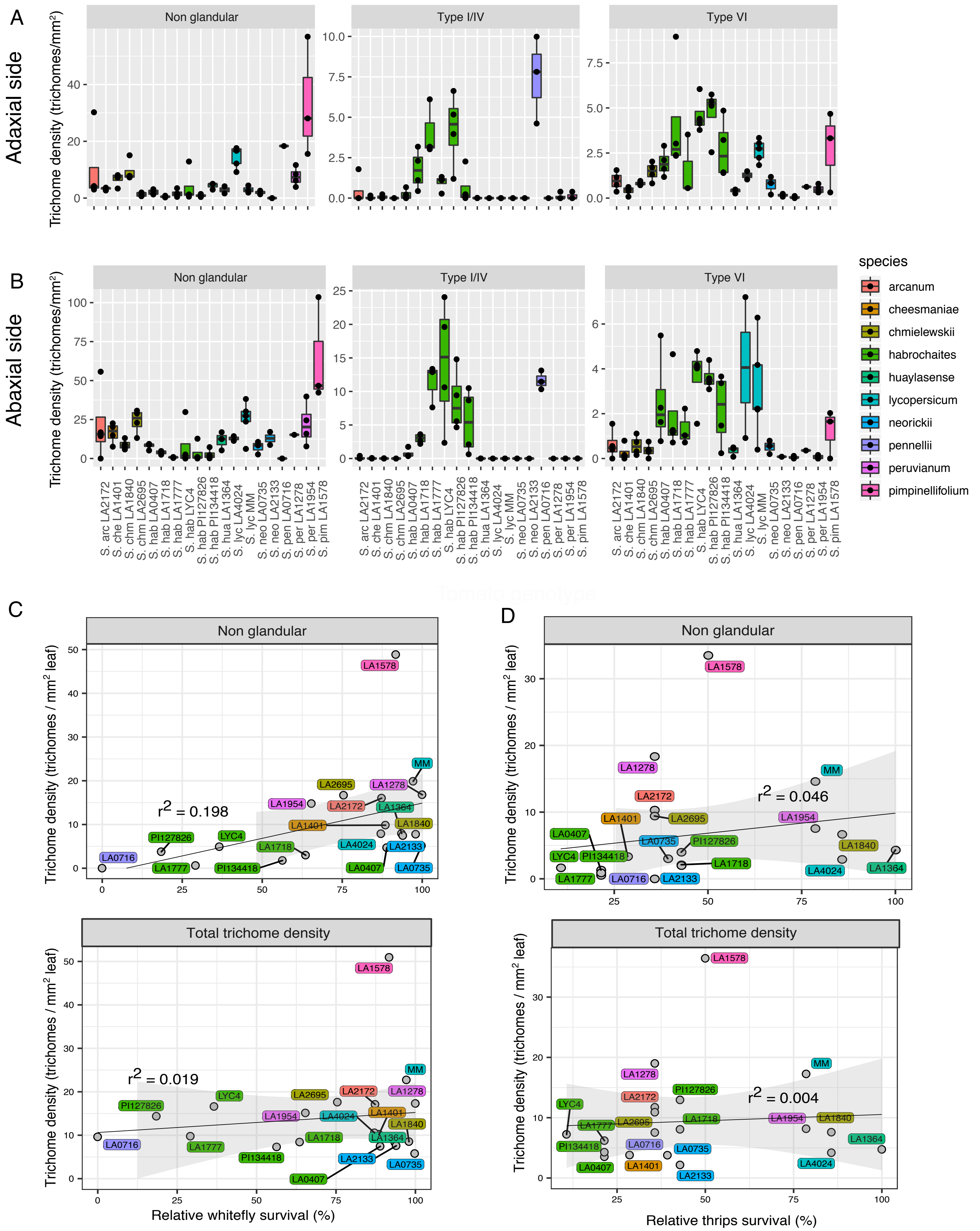

Additional file: Figure S3. Densities of non-glandular, type-I/IV and type-VI trichomes on the leaf surfance. The data supports Fig. 2 of the main text. (**A**) shows the densities on the adaxial side while the abaxial side is given in (**B**). Datapoints indicated by the black dots display the average density of two leaflets of one plant (n = 3-5). (**C-D**) Average non-glandular (top panel) and the average of the summed trichome types (bottom panel) versus the relative survival of whiteflies (**C**) and thrips (**D**). The densities of (**C**) includes both abaxial and adaxial suface, while (**D**) only includes densitites on the adaxial surface.
Non glandular trichomes consisted of summed types II, III, V and VIII. Accessions are colour-coded by spiecies.
