# Supplementary material for: Natural variation in wild tomato trichomes; selecting metabolites that contribute to insect resistance using a random forest approach
Source: BMC Plant Biol. 2021 Jul 2;21:315. doi: 10.1186/s12870-021-03070-x (PMC8252294; doi:10.1186/s12870-021-03070-x)
Supplement: Supplementary file 8 — Additional file 8: Figure S8. Mass spectra of metabolites with a significant feature importance that could not be verified by an authentic standard. Given spectra were used for identification by library comparison in combination with their Kovats Retention Index. [file 12870_2021_3070_MOESM8_ESM.pdf]

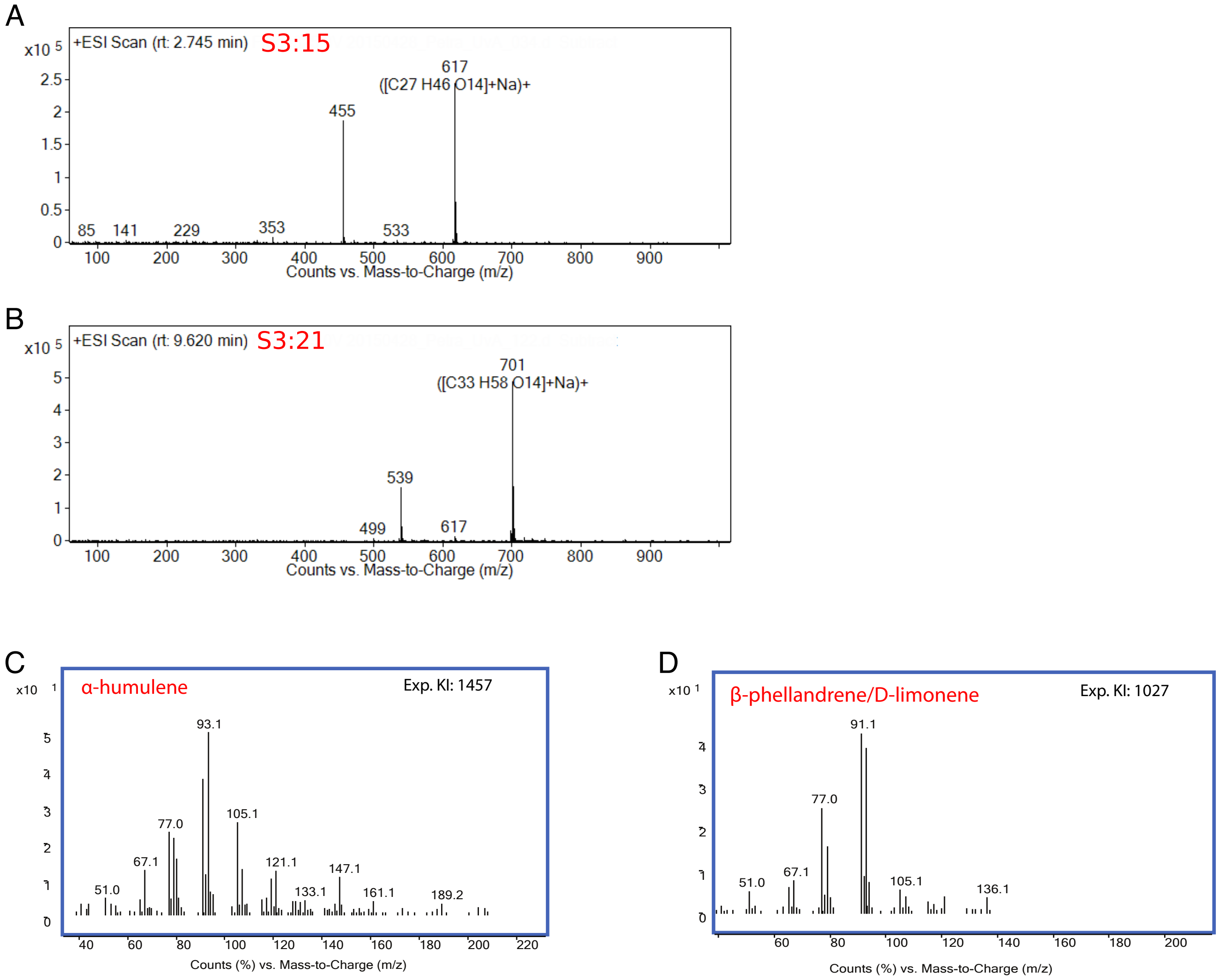

Additional file 8: Figure S8. Mass spectra of metabolites with significant feature importances that were not verified using analytical standards. This figure is supplemental to Table 2 of the main text.
(A, B) Acylsugars as measured by UHPLC-MS in positive mode. **(C-D)** Volatiles as measured by GC/MS-TOF. Metabolite identification is given in red. For the volatiles, the experimental Kovats Retention Index (KI) is given.
